# Thyroid Alert: Low Iodine and Perchlorate Effects in Women

**Published:** 2006-12

**Authors:** Richard Dahl

Perchlorate, an oxidizer in solid rocket fuel, is widely found in groundwater, drinking water, milk, vegetables, fruit, grain, and forage crops. Large doses of perchlorate have been shown to inhibit iodide uptake and reduce thyroid hormone production, which can contribute to metabolic problems in adults and abnormal neurodevelopment during gestation and infancy. Now, researchers at the CDC show that U.S. women with low iodine intake may be at risk for reduced thyroid function due to perchlorate exposure **[*EHP* 114:1865–1871; Blount et al.]**.

The researchers examined 2,299 men and women, aged 12 and older, who participated in the National Health and Nutrition Examination Survey. Examining the relationship between urine perchlorate concentrations and blood levels of the thyroid hormones thyroxine (T_4_) and thyroid-stimulating hormone (TSH), which stimulates T_4_, they observed that perchlorate was a significant predictor of thyroid hormone levels in women, but not in men.

Upon seeing this sex-based difference, the authors then categorized 1,111 women into “sufficient” and “low” iodine groups using a threshold of 100 μg/L urinary iodine, based on WHO recommendations. They found a slight relationship between perchlorate concentrations and TSH for the sufficient-iodine group, but a much stronger one for perchlorate and both T_4_ and TSH in the low-iodine group.

For the low-iodine group, higher perchlorate was associated with lower serum T_4_ and higher TSH. This relationship was consistent with what would be expected if perchlorate were inhibiting iodine uptake to such an extent that it interfered with thyroid hormone production. At the 50th percentile of urinary perchlorate (2.9 μg/L), the predicted decrease in T_4_ was 1.06 μg/dL; at the 95th percentile (13 μg/L), the predicted decrease in T_4_ was 1.64 μg/dL. Given that the normal range of T_4_ for women is 5–12 μg/dL, these predicted reductions were significant and indicate that even small increases in perchlorate exposure may inhibit the thyroid’s ability to absorb iodine.

In the United States, 36% of women have urinary iodine levels under 100 μg/L. In addition, the perchlorate doses seen to cause effects in this study are well below the 24.5 ppb reference dose recommended in 2005 by a National Academy of Sciences panel. The authors say that another large study is needed to confirm these findings; they are planning that study.

## Figures and Tables

**Figure:**
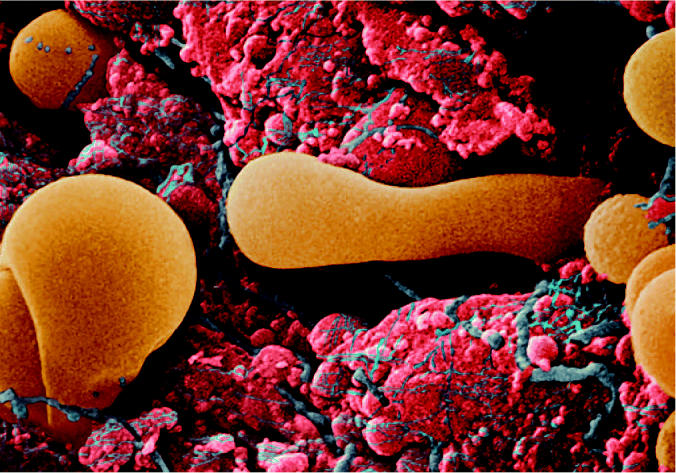
One thing leads to another Thyroid tissue, with thyroglobulin, a protein used to produce T_4_, shown in orange. Perchlorate may interfere with iodide uptake by the thyroid, leading to altered thyroid hormone synthesis.

